# The Clinicodemographic Features of Oral Squamous Cell Carcinoma: A Single‐Centre Retrospective Study

**DOI:** 10.1155/ijod/7540962

**Published:** 2026-06-29

**Authors:** Neetu Jain, Shashi Keshwar, Toniya Raut, Deependra Prasad Sarraf, Navin Agrawal

**Affiliations:** ^1^ Department of Oral Pathology, College of Dental Surgery, B.P. Koirala Institute of Health Sciences, Dharan, Nepal, bpkihs.edu; ^2^ Department of Clinical Pharmacology and Therapeutics, B.P. Koirala Institute of Health Sciences, Dharan, Nepal, bpkihs.edu; ^3^ Department of Conservative Dentistry and Endodontics, College of Dental Surgery, B.P. Koirala Institute of Health Sciences, Dharan, Nepal, bpkihs.edu

**Keywords:** Nepal, oral squamous cell carcinoma, retrospective, tobacco

## Abstract

**Introduction:**

In this retrospective study, a cohort from a Tertiary level Institute at Eastern Nepal was examined for clinicodemographic features of oral squamous cell carcinoma (OSCC).

**Materials and Methods:**

From 2014 to 2023, a total of 523 OSCC patients with histological confirmation were included. Descriptive statistics and chi‐square tests were used to assess socio‐demographic, habitual and clinicopathological data that were gathered from archival records.

**Results:**

The age group of 51–60 years old had the largest incidence of male patients (72.27%). Geographically, the Sunsari district accounted for most cases. Chewing smokeless tobacco (49.71%) was the most prevalent habit, lasting 10–20 years on average. The labial vestibule (37.2%) and the buccal vestibule (34.9%) were the most common main locations. Most lesions measured 2–4 cm and presented clinically as ulceroproliferative growths. At presentation, most lesions (68.45%) were painless.

**Conclusion:**

As per this study, in Eastern Nepal, OSCC primarily affects older men who have used smokeless tobacco in the past. It frequently manifests as painless ulceroproliferative lesions, which delays diagnosis. This hospital‐based study shows that OSCC frequently manifests as painless ulceroproliferative lesions and typically affects older males with long‐standing smokeless tobacco usage in Eastern Nepal. The results offer valuable regional clinicodemographic information that could help with tobacco control efforts, awareness campaigns and early clinical detection of suspicious oral lesions.

## 1. Introduction

In the year 2022, GLOBOCAN identified oral cancer (OC) as the sixteenth most prevalent cancer globally, with a total of 389,846 new cases and 188,438 fatalities. Notably, Asian nations represented nearly two‐thirds (65.8%) of all newly reported cases of OC [1]. Currently, new OC cases are mostly reported in developing countries [2]. It mainly affects the tongue and the floor of the mouth and is more common in males. Men who are middle‐aged to elderly are especially vulnerable to OC [3]. There is an increasing trend in the incidence of OC among people living in low‐ and middle‐income countries [4]. The global incidence of oral squamous cell carcinoma (OSCC) has been increasing in many countries, especially among younger people, with an expected rise of 30% in incidence each year by 2030. This increase is partly due to lifestyle changes, including higher alcohol consumption and tobacco use. Furthermore, the increasing occurrence of human papillomavirus (HPV)‐related oropharyngeal cancer also plays a role in this changing trend [2]. Unfortunately, the widespread practice of chewing betel quid and consuming tobacco and alcohol are important factors that make the Asian population more susceptible to OC [5].

Clinically, OSCC usually appears as ulcers, outward‐growing tumours, or areas of white (leukoplakia) or red (erythroplakia) patches [6]. Squamous cell carcinoma of the oral mucosa presents significant morbidity and mortality. Consequently, the 5‐year survival rate for conventional OSCC ranges from 75%–84%. However, in advanced clinical metastatic cases, there is around a 20% survival rate for the cases of the floor of the mouth and 36% for the cases located on the tongue [7].

OSCC is a disease that arises from multiple factors, including genetics, the environment, and molecular influences. Epidemiological studies are essential for understanding how often various cancers occur, to know their demographic characteristics and their incidence rates, as well as to pinpoint specific risk factors. Additionally, these studies provide valuable information about the effectiveness of various cancer control strategies [8]. Despite the fact that the primary demographic and clinicopathological data on OSCC can be similar across most studies, it is acknowledged that specific parameters can vary significantly between countries and even between regions within the same country. However, tobacco is considered the most important cause of OSCC [9]. It is also suggested that self‐reported oral health indicators, including frequency of routine dental examinations and frequency of tooth brushing, significantly affect survival in head and neck SCC [10, 11]. As tobacco consumption is a very common practice in Nepal, it is important to establish a relationship between tobacco consumption and the rate of OCs as well. According to the Nepal Development Research Institute, 27,100 people die annually owing to tobacco‐related diseases in Nepal, most commonly due to lung cancer [12]. Despite the high incidence of OC linked to tobacco use in South Asia, clinicodemographic information from Eastern Nepal is still scarce; the majority of Nepalese research studies that are currently accessible are either based on tiny datasets with inadequate clinicopathological characterisation or multicentric cancer registry reports. In addition to highlighting regional trends in smokeless tobacco use, lesion location, and clinical presentation, this study offers a 10‐year retrospective analysis of histologically proven OSCC cases from a tertiary referral centre in Eastern Nepal. Designing tailored awareness and tobacco control measures in populations with high smokeless tobacco consumption requires an understanding of these regional patterns.

The objectives of this study were to find out OSCC relation with gender, age, site, and habit (smokeless tobacco, smoking and alcohol intake) and OSCC clinical presentations (ulceration, nodular growth and size of the tumour). Thus, the aim of this study was to report the clinicodemographic aspects from a series of OSCC‐diagnosed cases in the Oral Pathology department at B.P. Koirala Institute of Health Sciences (BPKIHS), Nepal.

## 2. Materials and Methods

A retrospective study was conducted in the department of oral pathology at the College of Dental Surgery, BPKIHS, Nepal. This study included all cases of OSCC for which biopsies were submitted to the Department from 2014 to 2023. This research received approval from the Institutional Research Committee (IRC/2543/023) of BPKIHS.

This study included all the patients with a histologically confirmed diagnosis of OSCC and its variants, with an adequate case history and whose slides were properly appreciable with an adequate biopsy size and proper features for confirmation as OSCC. Patients with oral cavity tumours other than squamous cell carcinoma, patients having squamous cell carcinoma who underwent excisional biopsy or radical neck dissection, and the patients with recurrences of OSCC were excluded. The relevant data were collected from the requisition forms from the archives. The requisition forms were thoroughly reviewed to collect socio‐demographic information (age, sex and address), clinical details (lesion location, size, duration, clinical appearance and associated pain) and patient habits. The data were entered into Microsoft Excel 2010. Categorical variables were summarised through frequencies and percentages, while continuous variables were summarised through means and standard deviations. The chi‐square goodness of fit test, chi‐square independent test, and binominal tests were used for the statistical analysis using Statistical Package for the Social Sciences (SPSS) Version 24. The significance level was set at *p*  < 0.05.

## 3. Results

**Figure 1:**
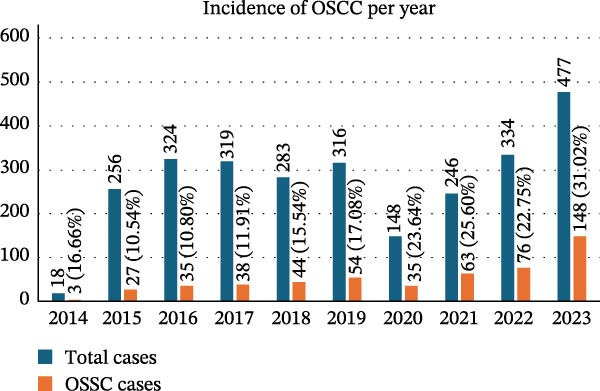
Incidence of OSCC from 2014 to 2023 (*n* = 523).

A total of 523 cases were analysed after meeting the inclusion criteria (Figure 1).

The socio‐demographic profile revealed that the patients were mainly male, commonly between 51–60 years old. However, the study also reported cases in younger people (20–30 years old). Geographically, Sunsari district had the highest number of cases, followed by Morang and Jhapa in the Koshi Province. The most prevalent habit was chewing tobacco, followed by a combined habit of chewing and smoking. The most common reported duration of habit was 10–20 years. However, intervals of less than 10 years to more than 50 years were also seen (Table 1).

**Table 1:** Socio‐demographic characteristics of the patients with OSCC (*n* = 523).

Variables		Number	Percentage
Gender	Male	**378**	**72.27**
	Female	145	27.72
Age category (years)	20–30	8	1.529
	31–40	55	10.516
	41–50	128	24.474
	**51–60**	**156**	**29.827**
	61–70	109	20.841
	71–80	53	10.133
	>80	14	2.676
Districts	Sunsari	**183**	34.99
	Morang	91	17.3999
	Jhapa	68	13.001
	Saptari	66	12.619
	Others	115	21.988
Habits	Tobacco chewing	**260**	49.71
	Tobacco chewing + tobacco smoking	104	19.885
	Tobacco smoking	36	6.883
	Tobacco chewing + alcohol	32	6.118
	Others	91	17.3999
Duration of habits (years)	<10	68	13.001
	**10–20**	**229**	43.785
	21–30	70	13.384
	31–40	67	12.81
	41–50	64	12.237
	>50	25	4.78

*Note:* Bold values are the most impactful values.

Based on clinicopathologic features, the labial and buccal vestibules were the most involved areas. Most of the lesions were 2–4 cm in size, while some cases also had lesions >6 cm. Ulceroproliferative growth was the most frequent clinical presentation. Pain or tenderness over the lesion site was noted in about 31% of patients. Although several patients presented after more than a year, the mean duration from the onset of the lesion to medical consultation was 1–3 months (Table 2).

**Table 2:** Clinical characteristics of the OSCC (*n* = 523).

Variables		Number	Percentage
Sites of the lesion	Buccal mucosa	9	1.72
	Floor of mouth	32	6.118
	Lip	13	2.485
	Palate	20	3.824
	Socket	3	0.573
	Tongue	68	13.001
	Buccal vestibule	**183**	**34.99**
	Labial vestibule	**195**	**37.284**
Size of the lesion	<2 cm	120	22.944
	**2–4 **cm	**271**	**51.816**
	>4–6 cm	86	16.443
	>6 cm	46	8.795
Appearance of the lesion	Growth	53	10.133
	Proliferative mass	25	4.78
	Red and white lesion	14	2.676
	Wound	21	4.015
	Ulcer	83	15.869
	Ulceroproliferative	**275**	**52.581**
	Non‐healing lesion	52	9.94
Pain/tenderness	Present	165	31.548
	Absent	**358**	**68.451**
Duration of the lesion (months)	** <1**	**146**	**27.915**
	1–3	193	36.902
	3–6	88	16.826
	6–12	54	10.325
	**>12**	**42**	**8.03**

*Note:* Bold values are the most impactful values.

## 4. Discussion

OC marks a significant global health alarm, and its incidence is associated to socio‐cultural habits [13].As per GLOBOCAN 2022, Asia accounts for 66.3 % (258440 cases), 62.4 % (683010 cases), and 75.1% (141465 cases) of incidence, prevalence and mortality rate for OC, respectively[1]. The present study reports a marked increase in incidence of OC from 16.7% to 31% from the years 2014 to 2023 (Figure 1). GLOBOCAN [1] 2022 also marks that, in Nepal, OCs ranked 7th most common cancer in incidence rate and 9th in death rate. As OC causes high mortality and morbidity, it is a concerning public health issue [2]. However, the necessity to address this expanding health issue is highlighted by the paucity of demonstrable data on OC in many settings, particularly in nations like Nepal. This study uses data from a tertiary hospital in eastern Nepal to fill the evidential gap. This dataset offers comprehensive information on clinical presentation, lesion sites, tobacco use patterns and symptom profile across a 10‐year period, in contrast to more general national registry‐based findings. This cohort’s high prevalence of vestibular involvement and smokeless tobacco use reflects regional behavioural patterns and could assist doctors in identifying high‐risk presentations typical of this area.

In the present study, Koshi Province (Table 1) accounted for the bulk of cases, which can be because the centre is in Koshi Province. The highest prevalence found in the districts of Sunsari, Morang and Jhapa (Table 1) might be due to dense populations, lower socioeconomic status and easier access to smokeless tobacco products. Not much data are available on the geographic distribution of OSCC cases around different provinces of Nepal. However, in a study by Poudel et al. [14] 20 % of patients were from Koshi province. The idea of ‘oral cancer belts’ in South Asia—localised areas where behavioural, occupational and environmental risk factors combine to form epidemiological hotspots for malignancies—is supported by this regional clustering. As per the literature review, OC is the most prevalent cancer in South and Southeast Asia as well as the Western Pacific islands of Bangladesh, India, Sri Lanka, and Papua New Guinea because of the use of smokeless tobacco and chewing areca nuts and behavioural, occupational and environmental risk factors [15].

It was noted in the present study that the condition was 2.6 times more common in males than in females (Table 1). This was consistent with the findings of Jaysooriya et al. [16]. They mentioned that men are disproportionately affected by OSCC in both developed (male to female ratio 2.5:1) and underdeveloped (male to female ratio 3:1) countries [16]. Reichal and Prethipa [17] also stated that compared to the female population (21.62%), the male population had a higher dominance of OSCC (78.3%). The conventional gender disparity in OC rates, which was characterised by a higher risk for men, was the result of a combination of factors, including the easier access to tobacco products for men and the strong social taboos against women engaging in such behaviours, particularly in countries such as India [17]. This disparity is currently being decreased. The cultural standards that previously restricted women from consuming tobacco and alcohol are gradually fading in numerous societies. As an increasing number of women from various age groups and socioeconomic statuses embrace these behaviours, a pattern already noted in developed countries, the traditional gap in risk factors is lessening [17, 18].

According to a study on OSCC in the South Indian population by Chamoli et al. [19], the disease is significantly more common in people above 40 years of age than in younger people. As per the literature, in terms of age, only 6% of OSCC patients are less than 45 years old, with most patients worldwide being older than 45 at initial diagnosis [19]. Similar findings were seen in the present study, where most of the patients were over 45 (Table 1). This may be brought on by the cumulative effects of tobacco smoking as well as ageing‐related immune system decline. However, we also observed cases in the age range of 20–30 (Table 1). This may be the result of young people being exposed to tobacco use at a young age since it is readily available [19]. Another cause of youngsters getting hooked to such habits can be a result of work pressure, stress and the endorsement of such products in movies and web series. However, this is an important finding that emphasises the importance of carefully examining every case with a suitable clinical presentation, regardless of age. Similar findings have also been reported in the study by Al‐Jamaei et al. [18], where they found an increase in the incidence of OSCC in the younger age group of 20–35 years.

According to a study by Sharma et al., [20], the buccal mucosa was the most common site of OSCC in Central Nepal. This was consistent with the findings of the investigation of Reichal and Prethipa [17] and associates. In contrast to prior studies where the buccal mucosa was the most prevalent location, particularly in South Asians, the tongue was the most widely prevalent site in the West [21]. However, the buccal and labial vestibule (53.2%) was the most common site of primary malignancy in the present study (Table 2). The present study results might be due to tobacco products’ placement within the buccal and labial vestibules that may cause alterations in these areas.

OSCC may manifest as an asymptomatic growth or ulcers. At the time of identification, lesions may manifest as erythroleukoplakias with central ulcerations, suggesting a suspicion of either invasive squamous cell carcinoma or severe dysplasia. However, in more severe cases, it typically manifests as ulceroproliferative growths with necrotic patches and extension to nearby tissues like muscle, bone and skin layers, along with metastases to the neck [22]. So, an indurated ulcer, an exophytic growth, an indurated non‐ulcerative patch (endophytic), or a combination of these characteristics are common presentation of OSCC [23]. In the present study, some cases appeared as a depressed ulcer with greyish‐white edges, elevated, everted, indurated borders and an infiltrated base and some as an exophytic, irregular lobulated lesion. However, most of the cases are represented as an ulceroproliferative growth (Table 2). Thus, oral ulcers can show simple to extremely complicated alterations that could indicate OC. When these lesions become more severe, suspicions are raised. As a result, proper diagnostic techniques are crucial, including the gold standard biopsy in addition to various non‐invasive techniques [24].

In the present study, ~70% of the patients did not experience any pain (Table 2). Ten to fifteen percent of patients in the present study postponed consultation for more than a year (Table 2), which greatly increased the chance of malignant development, even though the majority (~60%) sought medical counsel within one to 3 months after discovering symptoms. This asymptomatic presentation is the major cause of diagnostic delay. It is observed that over 50% of OSCC patients in rural South Asia are diagnosed at a late stage due to a combination of poverty, lack of awareness, and accessibility constraints, supporting this delay [17]. As OSCC lesions develop, their initial size might vary from a few millimetres to several centimetres, which is also related to the prognosis of the case [22]. In the present study, the size of most of the lesions was between 2–4 cm; however, we also reported the number of cases with a size of more than 6 cm (Table 2).

Even though OSCC prevalence and related morbidity and mortality vary greatly throughout the world, they are higher in regions where tobacco and areca nut use are prevalent [21]. Dwivedi et al. and Ray et al. [25, 26] observed that the most common type of tobacco was smokeless tobacco, mainly in the form of gutka, followed by smoked tobacco, such as bid. The results of the present study also showed that smokeless tobacco is the most widely used type, followed by smoked tobacco (Table 1). In the present study, the duration of habit in most of the cases was above 5 years (Table 1). It is believed that about 42% of cases of OC are caused by smokeless tobacco alone, mostly because of the significant amount of reactive oxygen species released during chewing [27]. Because of the combined effects of alcohol and tobacco, people who use both have a much higher chance of developing OSCC. Because alcohol dehydrates cell membranes, tobacco’s carcinogens can more easily penetrate oral tissues [27].

The use of tobacco results in hundreds of billions of dollars in economic damage and nearly 6.4 million deaths annually worldwide. If current trends continue, tobacco use will kill over 8 million people annually by 2030 [28]. Most people continue to smoke despite being aware of the negative health effects of tobacco use and carcinogens, which can infiltrate the body’s multiple systems [28]. Numerous substances go into making a cigarette, and some tobacco companies may add flavours to their products to make them more appealing, even though they could be unhealthy [28]. Approximately 90% of the cases of OC that arise in South‐East Asia each year are caused by chewing and smoking behaviours. Depending on the product, tobacco may contain over 60 known or suspected carcinogens that can raise the relative risk of cancer through a variety of mechanisms, such as immune system effects, lipids, carbohydrates and DNA that disrupt cell cycle‐regulated mutations, oxidative stress on tissues, and persistent reactive oxygen species [29]. Lifestyle, environmental, and occupational factors that are connected to socio‐demographic variables are examples of additional risk factors [30–33]. Additionally, hereditary variables affecting cancer immunosuppression, etc., may also be involved [15, 28, 34, 35]. Poor food and nutrition, as well as obesity, are also considerable risk factors for OCs [36]. OC susceptibility has also been demonstrated to be increased by inadequate dental care and chronic intraoral inflammation, including periodontitis, chronic mechanical stress, and recurrent oral ulcers [15, 34, 37, 38]. The HPV subtypes 16, 18, 31, 33, and 35 are also found to be associated with oral carcinogenesis among the various infection types [39]. UV radiation is one type of environmental exposure that can result in lip cancer [39].

## 5. Conclusion

OC is a serious and growing public health concern, especially in Eastern Nepal and throughout South Asia. This data support a troubling local increase in incidence, which is like the regional burden mostly caused by the high prevalence of smokeless tobacco. Males and older adults are disproportionately affected by the condition, while its concerning prevalence in younger populations points to a concerning trend. Early lesions are frequently asymptomatic, and patients frequently put off seeking care, which results in late‐stage diagnosis and increased mortality. These results highlight the critical need for mass awareness campaigns to encourage early diagnosis, targeted public health measures aimed at tobacco reduction, and the requirement that all worrisome oral lesions, regardless of age, get a comprehensive clinical evaluation.

The strengths of this study were the relatively high sample size, histological confirmation of all included cases, and the 10‐year study duration, which permitted investigation of regional clinicodemographic patterns in Eastern Nepal.

## 6. Limitations

The results of this hospital‐based retrospective study from a single tertiary care facility have limited generalisability as it could not be entirely applicable to the Nepalese population because it mainly gathers information from individuals who can afford and have access to specialist medical care, which also leads to selection bias. Furthermore, a more thorough knowledge of the risk profiles might have been possible if there had been more extensive information available on the use of tobacco products, dietary practices, or genetic factors. It also lacks the presentation of different ethnic and cultural backgrounds. It cannot determine the true incidence of OC in Nepal.

**Nomenclature**
BPKIHS: B.P. Koirala Institute of Health Sciences
DNA: Deoxyribonucleic acid
GLOBOCAN: Global Cancer Observatory
HPV: Human papilloma virus
OC: Oral cancer
OSCC: Oral squamous cell carcinoma
SCC: Squamous cell carcinoma
SPSS: Statistical Package for the Social Sciences.

## Author Contributions

**Neetu Jain**: conceptualisation, data curation, investigation, formal analysis, writing – original draft, writing – review and editing. **Shashi Keshwar**: formal analysis, validation, writing – review and editing. **Toniya Raut**: data curation, writing – review and editing. **Deependra Prasad Sarraf and Navin Agrawal**: writing – review and editing.

## Funding

This study did not receive any specific funding.

## Disclosure

All authors have read and approved the final version of the manuscript. Corresponding author (Neetu Jain) had full access to all of the data in this study and takes complete responsibility for the integrity of the data and the accuracy of the data analysis.

## Ethics Statement

The study has been approved by the Institutional Research Committee (IRC/2543/023) of B.P Koirala Institute of Health Sciences, Dharan, Nepal.

## Consent

Written informed consent has been taken from the participant.

## Conflicts of Interest

The authors declare no conflicts of interest.

## Data Availability

The authors confirm that the data supporting the findings of this study are available within the article.
